# Bacterial L-forms: Key Mechanisms of Drug Resistance, Disease Recurrence, and Immune Evasion

**DOI:** 10.1007/s12088-025-01522-8

**Published:** 2025-09-22

**Authors:** Songling Lei, Dan Chen, Junliang Wu, Kewang Hu, Mingli Zhu

**Affiliations:** Hangzhou Xixi Hospital Affiliated to Zhejiang Chinese Medical University, Hangzhou, People’s Republic of China

**Keywords:** Bacterial L-form, Cell wall–deficient bacteria, Bacterial resistance, Infectious disease, Cell wall, Immune system

## Abstract

**Abstract:**

Bacteria enter the L-form state after shedding their cell walls in response to adverse conditions for survival; these bacteria can revert to their walled morphology after the unfavorable factors are removed. Bacterial L-forms can be either unstable (typically observed in clinically isolated and induced L-forms) or stable (associated with two specific mutation types). In recent years, bacterial L-forms have been implicated in antibiotic resistance, disease recurrence, and origin of life. However, because identifying bacterial L-forms is difficult, they are often overlooked in clinical settings. In this review, we elucidate the relationships of bacterial L-forms with their cell walls, mutation, proliferation, antibiotic resistance, and disease recurrence, as well as with the host immune system. We also highlight the challenges and future prospects for bacterial L-form research.

**Graphical Abstract:**

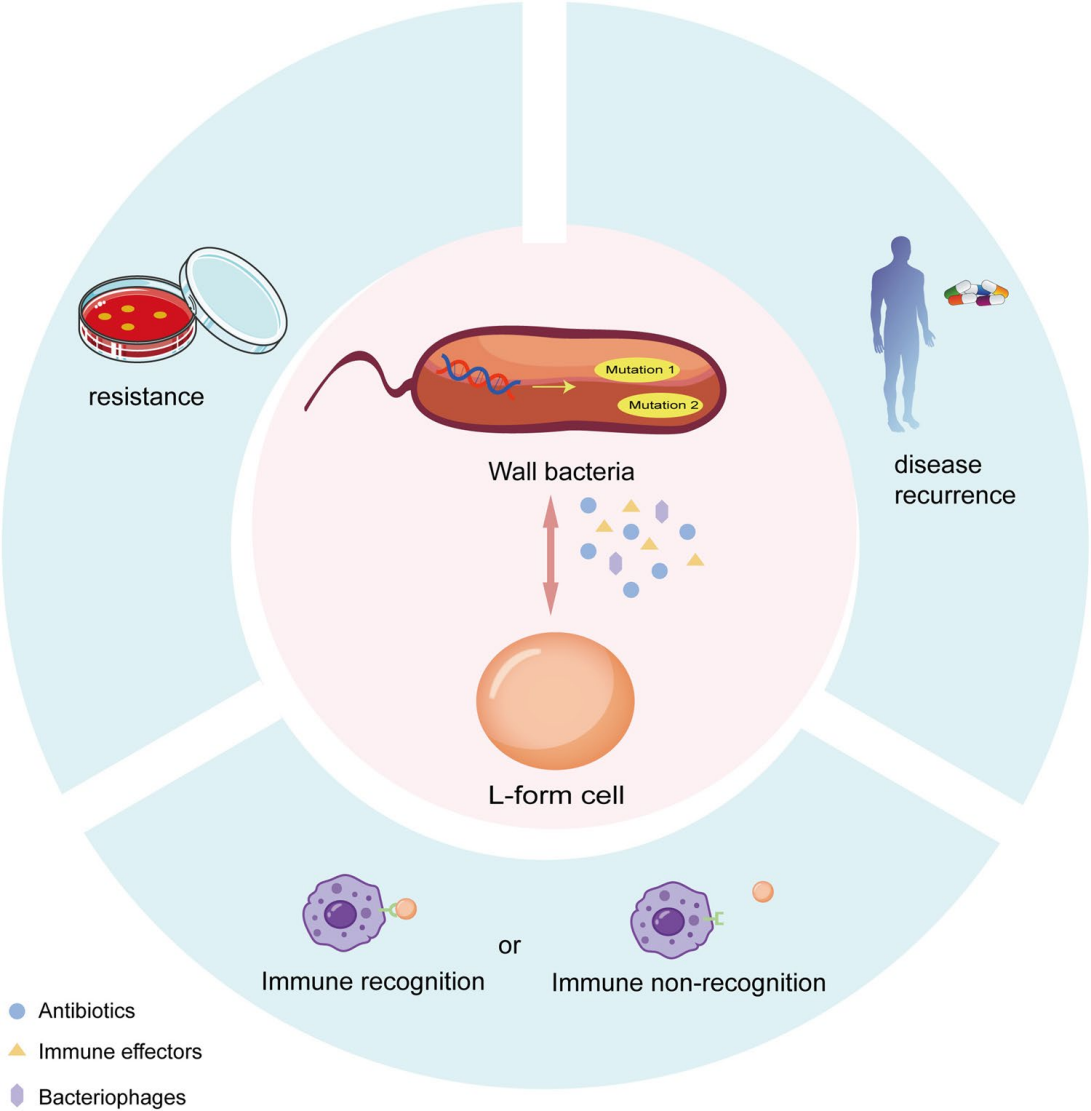

## Introduction

The cell wall is a structural component present in most bacteria except mycoplasma, which aids in protecting bacterial cells against hypoosmotic conditions, maintaining their morphology, and regulating material exchange between their interior and exterior; as such, the cell wall is considered a target site for various antimicrobial agents [1–4]. Klieneberger discovered bacteria lacking a cell wall for the first time in 1935; in particular, the author observed *Streptobacillus moniliformis* lacking a cell wall in an aged culture of the bacterium and named them bacterial L-forms (where “L” refers to the first letter of the research institute where the discovery was made). Since then, bacterial L-forms have also been referred to as L-phase, L-stage, L-variant, L-organisms, and cell wall–deficient forms [5, 6]. Subsequently, several studies reported that under unfavorable conditions with osmotic protection, various bacteria, such as *Escherichia coli*, *Staphylococcus aureus*, *Mycobacterium tuberculosis*, *Salmonella typhi*, *Listeria monocytogenes*, *Bacillus subtilis*, and *Streptomyces* [7–15], can shed their cell walls to form round or irregularly shaped bacterial L-forms. However, distinguishing these L-forms from protoplasts and dormant bacteria is crucial. Although protoplasts also lack cell walls, they cannot proliferate as L-forms do [16]. As proposed by Mark et al. [17] in 2024, bacterial dormancy can be universally defined as a temporary adaptive state marked by reduced metabolic activity and prolonged growth arrest, allowing bacterial cells to survive under adverse conditions. Similar to dormant bacterial cells, bacterial L-forms facilitate bacterial survival under adverse conditions; however, the L-forms remain metabolically active and continue to grow. Gram-positive and -negative bacterial L-forms demonstrate higher adenosine triphosphate (ATP) levels than their walled counterparts; therefore, bacteria L-forms remain metabolically active [18]. In contrast, bacterial L-forms demonstrate lower growth rates and pathogenicity than their walled counterparts [19]. With technological advancements, the entire process of bacterial cell transition between the walled state and the L-form state (and vice versa) after the removal of adverse conditions can be observed through confocal-laser-scanning, time-lapse, or phase-contrast microscopy. The L-form transition can also be observed in live zebrafish larvae through phase-contrast or fluorescence microscopy [20]. In recent years, bacterial L-forms have been implicated in bacterial drug resistance [21], disease recurrence, and origin of life [16, 20]. In this review, we elucidate the generation and proliferation of bacterial L-forms, as well as their relationship with antibiotic resistance, disease recurrence, and host immune system.

## Relationship between cell wall and bacterial L-form

Bacteria are categorized as gram-positive and gram-negative according to the structure of their cell walls, composed of peptidoglycan (PG) scaffolds crosslinked by short peptides. [22]. In the first step of PG synthesis, fructose-6-phosphate (F-6-P) is synthesized through the hexosamine pathway to form uridine diphosphate *N*-acetylglucosamine (UDP-GlcNAc). UDP-GlcNAc is then converted to uridine diphosphate-*N*-acetylmuramic acid (UDP-MurNAc) via *MurA* and *MurB*. Subsequently, five amino acids are linked to form uridine diphosphate *N*-acetylmuramyl pentapeptide, which then combines with undecaprenyl phosphate (UndP) to produce lipid I under *MraY* catalysis. Finally, uridine diphosphate *N*-acetylglucosamine is transferred to lipid I by *MurG* to form lipid II. All the aforementioned steps occur at the cytoplasmic membrane. Lipid II is then flipped from the inner to the outer side of the cell membrane by the enzyme *MurJ*, a process driven by a proton motive force [23]. After lipid II moves to the outer side of the cytoplasmic membrane, it is polymerized into a PG layer by glycosyltransferase and transpeptidase. The resulting UndP then returns to the inner side of the cell membrane to bind with newly synthesized lipid II via a process called UndP recycling. This cyclical process results in peptidoglycan scaffold formation [24, 25]. Proteins assisting in flipping lipid II from the inner to the outer side of the cell membrane remained unclear until recently when Ian et al. [26] discovered two widely conserved protein families that can influence UndP recycling: Undecaprenyl phosphate transporter A (UptA; a DedA superfamily member) and polyprenyl-phosphate transporter (PopT; containing the DUF368 domain). The authors also indicated that the recycling function is widely conserved within these two protein families.

Under adverse conditions, including exposure to cell wall–targeting antibiotics [7, 13], immune effectors [27], bacteriophages [28], or extreme nutrient depletion during culture [29], bacteria may shed their cell walls, forming bacterial L-forms [16]. Under normalized hypertonic conditions, some bacterial L-forms undergo reversion to the wall-replete state, detectable by electron microscopy[20]. Although a precise definition of bacterial L-forms remains unavailable, bacteria initially possessing a cell wall but surviving after losing it due to various adverse factors are typically referred to as bacterial L-forms. Based on revertible capacity to walled states, these L-forms are subdividable into stable and unstable categories [16]. Bacterial L-forms do not entirely lose their cell walls. In some studies, the L-forms have been noted to retain small amounts of PG residues, and PG synthesis is dynamic and variable in L-forms [30–32]. In the laboratory, wall-targeting antibiotics are commonly used to induce bacterial L-forms. For example, 0.5 ppm of lysozyme and 64 ppm of meropenem can induce *Escherichia coli* and *Bacillus subtilis* to transition into bacterial L-forms[18]. Alternatively, bacterial L-forms can be induced using penicillin on a hypertonic culture medium[33].

## Mutation and Proliferation of Bacterial L-form

### Stable and Unstable L-forms

**Fig. 1:**
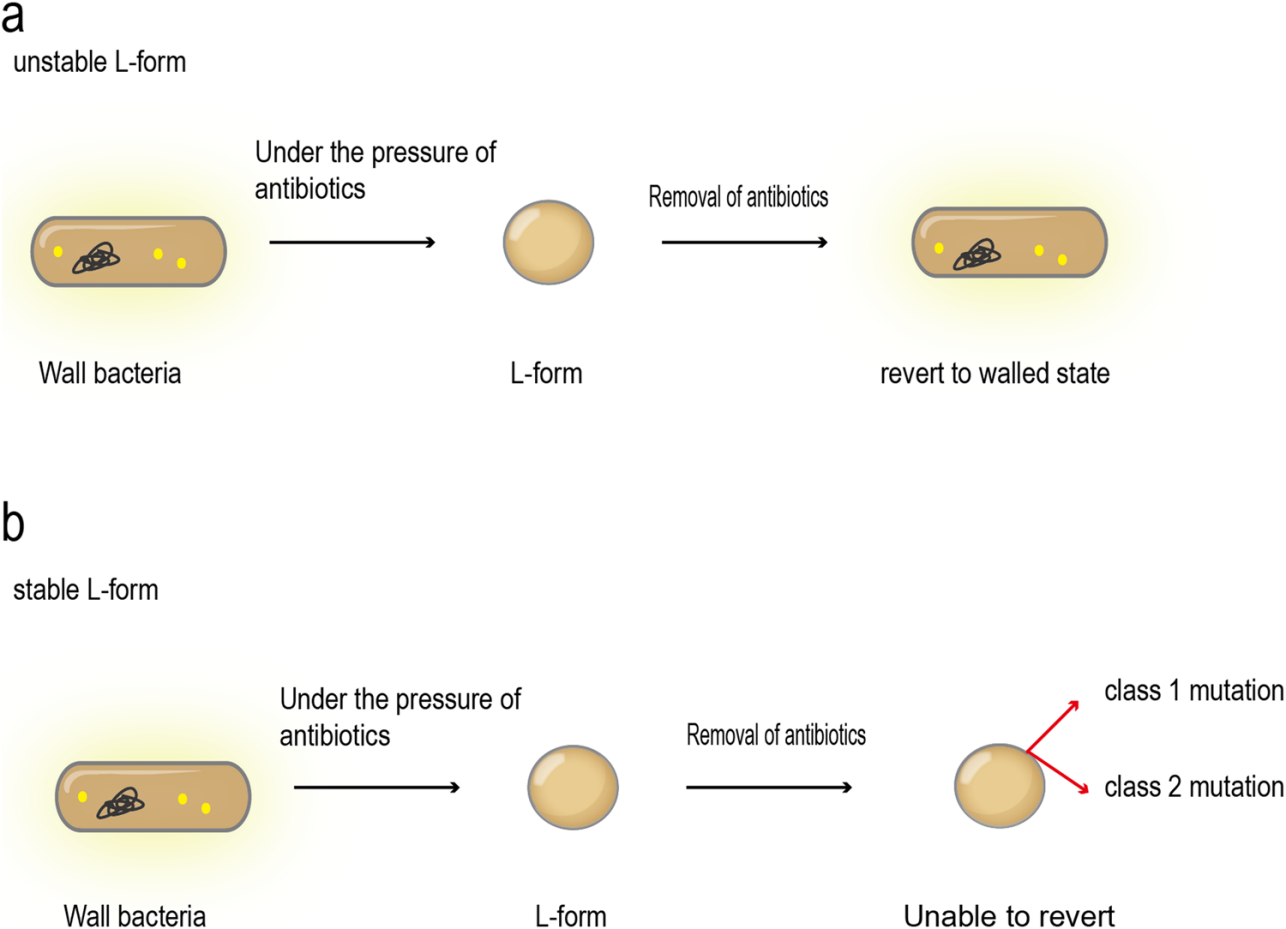
**a** Unstable bacterial L-form. A walled bacterial cell transitions to L-forms under the pressure of an antibiotic but reverts to its walled morphology when the antibiotic is withdrawn. **b** Stable bacterial L-form. A walled bacterial cell transitions to L-forms under the pressure of an antibiotic but does not revert to the walled state after antibiotic removal. Acquiring a stable L-form requires at least two mutations: one that leads to excessive fatty acid synthesis and another that counteracts the increase in reactive oxygen species production

As mentioned above, these L-forms are subdividable into stable and unstable categories [34] (Fig. 1). The stable L-forms of bacteria is defined as a state in which bacteria, even after the removal of the adverse factors that initially induced the L-form transition, remain in a wall-deficient state and cannot revert to their original cell wall structure. Stable L-forms typically arise due to genetic mutations in bacteria that have been subjected to persistent adverse conditions or as a result of artificial gene knockouts targeting relevant genes. [6, 34]. In contrast, unstable L-form bacteria refer to those that transform into L-forms under unfavorable conditions, but can revert to their original walled morphology once they are removed from the adverse environment (such as after antibiotic exposure)[35]. However, unstable L-form bacteria have the potential to transition into stable L-forms. When initially exposed to adverse conditions, these bacteria enter an unstable L-form state. If these unfavorable conditions persist, genetic mutations may eventually occur, leading to the formation of stable L-forms that enable the bacteria to better cope with the ongoing harsh environment [34, 35]. The L-forms isolated in clinical settings are usually unstable L-forms that are capable of reverting to their walled morphology. [20]. Supplementary imaging provided by Alexander et al. [31] presents the entire process of *E. coli* transitioning from a rod shape to an unstable L-form. Bacterial L-forms and walled cells exhibit minimal differences at the protein level but significant differences at the metabolic level. Thus, bacterial L-forms primarily regulate their survival in a wall-less state through metabolic pathways. Gene sequencing revealed that compared with their normal walled counterparts, the unstable L-forms of both *E. coli* and *S. aureus* demonstrate differences in expression of genes related to envelope stress, DNA repair, iron homeostasis, membrane biosynthesis, and drug efflux [33, 35]. Additional in-depth studies on unstable L-forms of *S. aureus* have revealed additional differences in gene expression related to metabolism, regulation, phage-related processes, virulence, transport, stress responses, and RNA metabolism [10]. In the screening of a mutant library for unstable *S. aureus* L-forms, mutations were identified in *glpF* (encoding a protein involved in glycerol uptake) [36] and *glpK* (encoding glycerol kinase) [37]. Furthermore, compared with those in their walled counterparts, the unstable *S. aureus* L-forms demonstrated 144- and 68-fold higher expression levels of the aforementioned genes, respectively. Moreover, adding low concentrations (1%) of glycerol to the medium significantly enhanced the transition of *glpF* mutants to L-forms. Thus, glycerol assimilation may be crucial in L-form generation[38]. Some studies have compared the gene expression levels between unstable bacterial L-forms and their walled parental counterparts. However, other studies suggest that there are almost no genetic changes in unstable bacterial L-forms. This discrepancy could be due to variations in the duration of L-form induction or differences in antibiotic dosages, and which may place unstable bacterial L-forms at different stages of gradual genetic mutation or at a stage where no mutation has occurred. In the study where genetic mutations were virtually non-existent, the L-forms underwent reversion to morphologically intact progenitors rapidly, regaining their cell wall within 20 h after antibiotic removal. This suggests that the reversal of L-forms to their morphologically intact progenitors may depend on whether genetic mutations have occurred and the extent of such mutations. It may also be regulated by the degree of unfavorable environmental stress. When the level of stress is high, genetic mutations are more likely to occur, whereas under lower stress levels, the likelihood of mutations may be reduced. Moreover, *S. aureus* L-forms can form distinct β-hemolytic zones on media containing penicillin at high concentrations. These L-forms possess fewer negative charges than their walled counterparts; moreover, the zeta potential differs between the two cell types. This results in enhanced aggregation of bacterial L-forms. Moreover, the egg-like colonies of bacterial L-forms on a solid medium originate from single progenitor cells. The occurrence of bubbling, irregular budding, or external vesicles may be a proliferation mechanism of these unstable bacterial L-forms [10].

The inherent instability of bacterial L-forms, however, renders them unsuitable for experimentation by using contemporary molecular biology techniques. As such, Leaver et al. sought to identify a method through which unstable bacterial L-forms can be transformed into stable variants. The authors discovered that by inhibiting the *Pxyl* promoter, which controls the *murE* operon, followed by introducing a second copy of the repressor gene *xylR* and subsequently applying penicillin selection, stable L-forms of *B. subtilis* can be obtained. Furthermore, the authors discovered that a single-point mutation in *ispA*, which encodes squalene synthase, can aid in achieving stable bacterial L-forms [11]. Mercier et al. proposed that the *ispA* mutation stabilizes L-forms undergoing morphological changes [39]. In contrast, Kawai et al. [40] demonstrated that the *ispA* mutation affects L-form growth by reducing electron transport chain activity. Moreover, the effects of the *ispA* single-point mutation can be substituted by mutations in other genes.

**Fig. 2:**
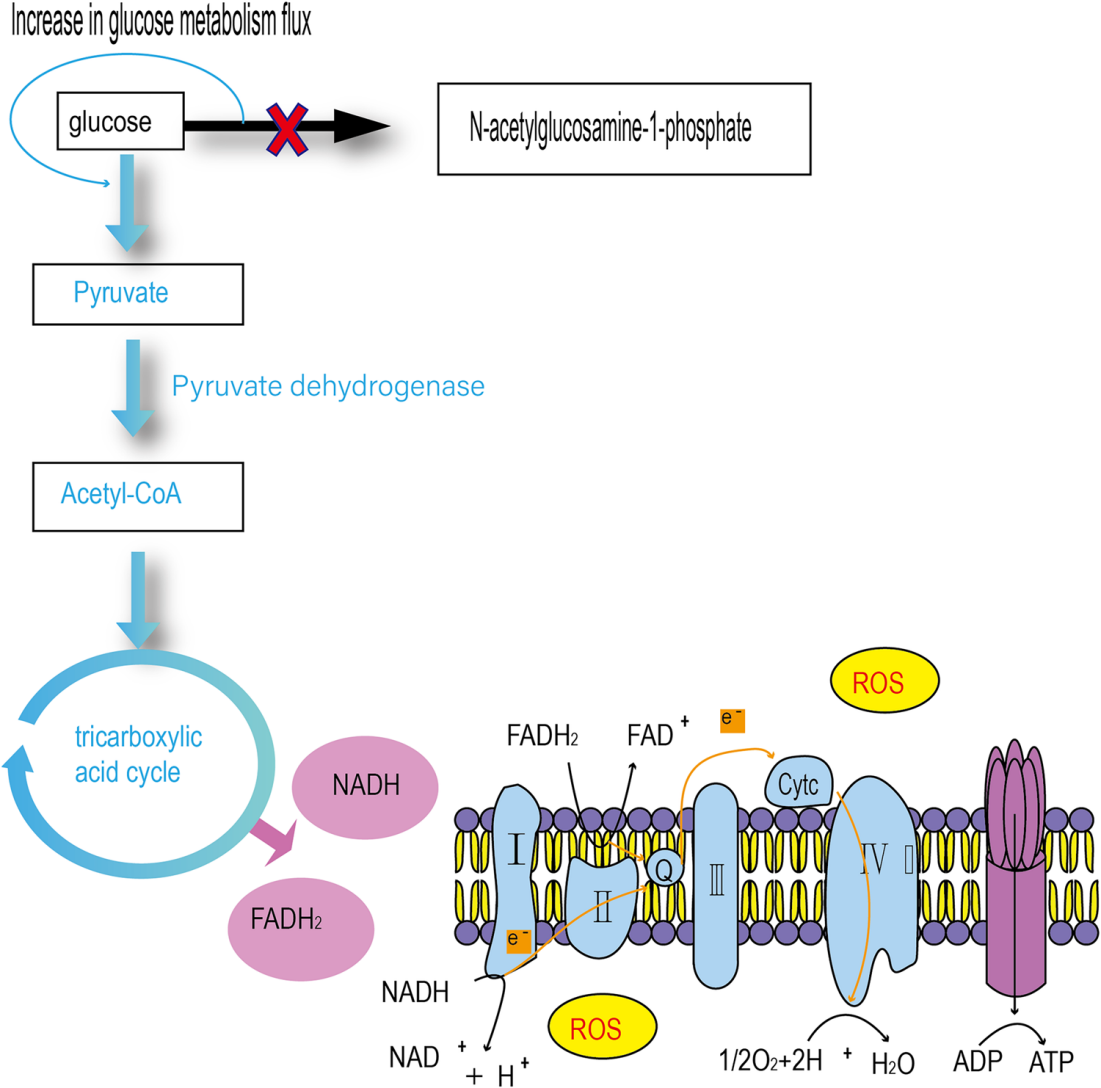
When *N*-acetylglucosamine-1-phosphate synthesis from glucose is inhibited, excess glucose enters the glycolytic pathway, leading to increased pyruvate production. Pyruvate is converted into acetyl coenzyme A via pyruvate dehydrogenase. Acetyl coenzyme A then enters the TCA cycle, generating NADH and FADH2—both of which facilitate electron transfer in the respiratory chain and ultimately lead to ATP and H_2_O production. Simultaneously, this process leads to an increase in the generation of ROS as respiratory chain byproducts

Mercier et al. also proposed that stable bacterial L-form acquisition requires at least two genetic mutation types. One of these mutations increases fatty acid synthesis [39]. Correspondingly, mutations resulting in reduced fatty acid synthesis can restore the growth of cells with defects in PG synthesis [41]. The other type of mutation counteracts the increase in reactive oxygen species (ROS). Kawai et al. [42] found that ROS production, which is harmful to cells, is increased in protoplasts; this is because the precursor of PG synthesis, *N*-acetylglucosamine-1-phosphate, is synthesized from glucose, and a blockage of PG synthesis leads to an increase in the flux of glucose metabolism, leading to an increase in the amount of pyruvic acid, a glycolytic pathway product. Pyruvate can, in turn, be converted to acetyl coenzyme A under the action of pyruvate dehydrogenase to enter the tricarboxylic acid (TCA) cycle, which leads to the production of the respiratory chain carriers nicotinamide adenine dinucleotide (NADH) and flavin adenine dinucleotide (FADH2). These carriers facilitate the transfer of electrons in the respiratory chain, which are ultimately shuttled back to Complex IV via cytochrome c to provide the electrons for the eventual reduction in oxygen, generating ATP and H_2_O [43]. Concurrently, this process produces ROS as a byproduct of the respiratory chain (Fig. 2). Bacterial L-forms have relatively low ROS levels because mutations reduce ROS production via the respiratory chain. Artificial addition of ROS scavengers or anaerobic cultivation can also promote L-forms growth [40, 44]. Metabolomic analyses have revealed lower levels of reduced glutathione in *Escherichia coli* L-forms than their walled counterparts; moreover, the glutathione system is necessary for cellular resistance to oxidative stress [45]. However, the reductions in glutathione levels may be attributable to increases in bacterial oxidative stress [31].

A study performed genome sequencing on two stable *E. coli* L-form strains and discovered that several genes considered essential for bacterial growth in walled bacteria had undergone mutations in these strains. These included genes involved in PG synthesis (e.g., *murC* and *mraY*), target genes of β-lactam antibiotics (e.g., *mrcA* and *mrcB*), genes essential for fatty acid synthesis (e.g., *fabI*), and genes essential for the oxidative phosphorylation pathway and the base excision repair pathway [44].

Both stable and unstable bacteria L-forms exhibit a common characteristic: cell wall shedding to survive in an unfavorable environment. However, the loss of protection afforded by a cell wall makes them more susceptible to oxidative stress. To counteract the oxidative stress damage by maintaining cellular redox balance, bacteria L-forms demonstrate upregulated expression of genes encoding antioxidant enzymes (e.g., catalase and superoxide dismutase) and genes associated with oxidative stress response (e.g., *arcB*, *ompC*, and *sosR*) [46]. Increased ROS production can even lead to the upregulation of genes related to drug efflux pumps, resulting in enhanced resistance to multiple antibiotics [47]. Unfavorable conditions and oxidative stress also damage bacterial DNA, which the affected bacteria mitigate through upregulation of DNA repair-related genes (e.g., *recA* and *walR*). Moreover, bacteria L-forms generate more ATP to meet the increased energy demands required for survival under adverse conditions. Analysis of gene expression in bacterial L-forms revealed a reduction in the expression of cell division–related genes (e.g., *ftsW*, *ftsL*, *ftsA*, *ftsZ*, and *divIB*), whereas growth-related genes are upregulated. This results in continuous bacterial growth without cell division, resulting in cellular enlargement. In unstable bacteria L-forms, genes involved in cell wall synthesis, such as *murG*, *murD*, *murB*, and *ispA*, are also upregulated. This may explain the ability of unstable L-forms to resume a walled morphology after the removal of unfavorable factors. Therefore, the ability of bacterial L-forms to survive in hostile environments and the consequent enhancement of antibiotic resistance results from the synergistic effects of cell wall synthesis, cell division, metabolic alterations, and stress responses [18].

### Bacterial L-form Proliferation

**Fig. 3:**
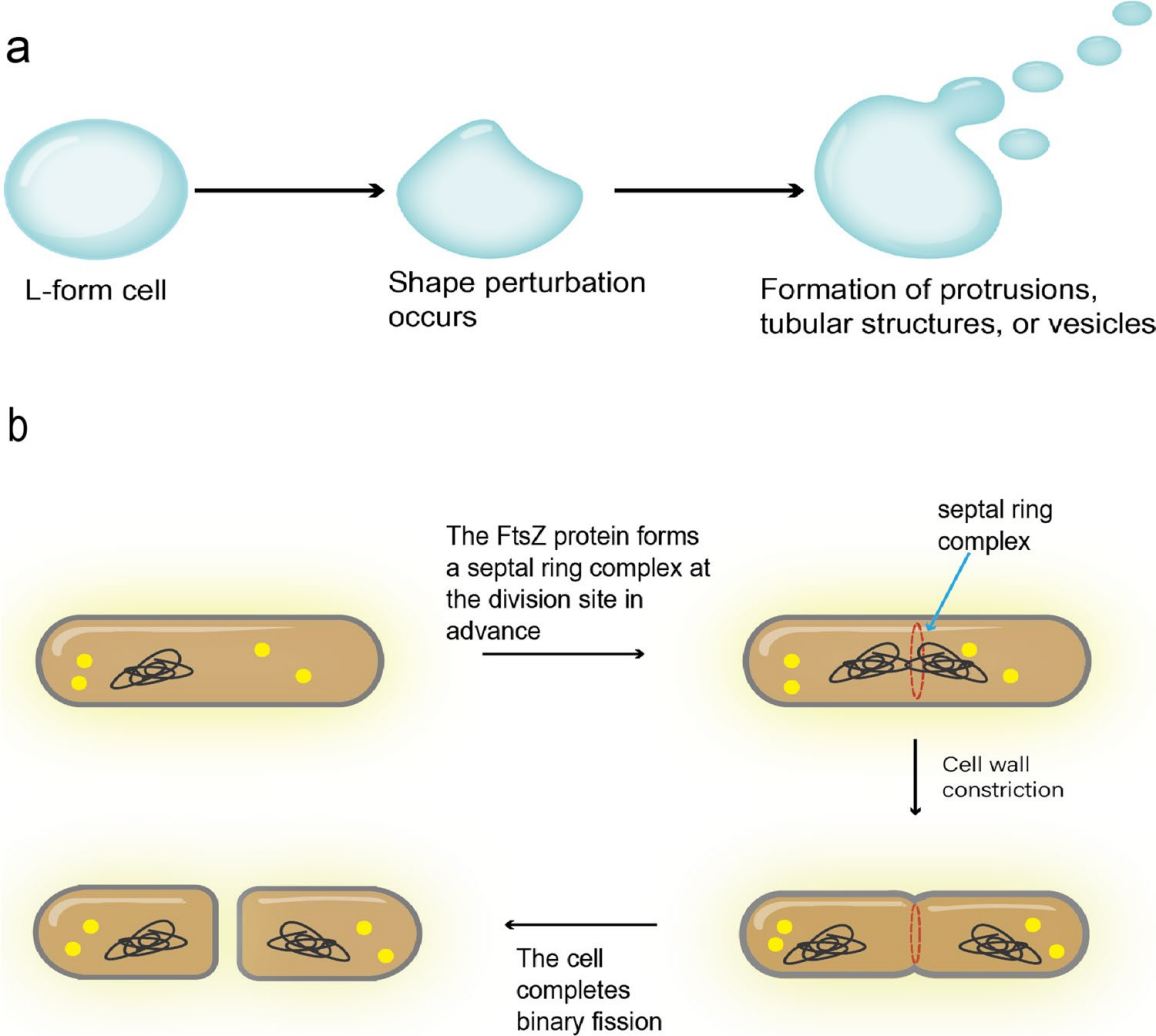
**a** Mode of proliferation in bacterial L-forms. L-forms undergo shape perturbations, followed by protrusion, tubule, or vesicle formation, with vesicle-like structures eventually detaching from the parent cell. **b** Mode of proliferation in walled bacterial cells. FtsZ protein first assembles into a dense Z-ring at the division site, which further develops into a septal ring complex. Subsequently, the cell wall constricts, followed by binary fission

Bacterial L-forms can proliferate through a process called vesicle scission (Fig. 3) [21], a mechanism associated with alterations in membrane synthesis rates. Impairment of peptidoglycan precursor synthesis may induce excessive membrane production via unknown mechanisms. Under an electron microscope, shape disturbances can be observed in bacterial L-forms, followed by the emergence of protrusions, tubular structures, or vesicles. Eventually, vesicle-like structures detach from the parent body [11, 31]. In contrast to binary fission observed in walled bacteria, bacterial L-form proliferation does not rely on the FtsZ system [11]. Walled cells of most bacteria (except *Streptococcus pneumoniae* [48]) divide through the FtsZ system. FtsZ is a tubulin homolog widely present in walled bacterial cells, which forms a dense Z-ring comprising treadmilling filaments at the division site early in the process. This Z-ring recruits other proteins essential for cell division, assembling into the divisome and further forming a septal ring complex. Subsequently, the cell wall constricts, followed by the completion of binary fission (Fig. 3b) [49–51]. However, some studies have shown that Z-rings can be observed in bacterial L-forms. This may be because these L-forms are in the process of reverting to their walled state. [52]. However, bacterial L-form proliferation is driven by changes in the surface area-to-volume ratio, resulting from increased fatty acid synthesis [39]. Fatty acid accumulation enhances membrane fluidity, promoting L-form division. In contrast, compounds such as demurilactone A and manidipine can inhibit L-form division by reducing membrane fluidity, leading bacterial L-forms to enter an expanded, rounded state resembling a terminal stage in which division is impossible [9, 21].

The two aforementioned studies indicated that bacterial L-form proliferation is associated with increased fatty acid synthesis and enhanced membrane fluidity. They also provided novel insights into potential clinical approaches for treating recurrent infections caused by bacterial L-forms. However, additional experiments substantiating these conclusions further are warranted. Moreover, the proliferation of gram-negative bacterial L-forms may be related to outer membrane expansion [53].

## L-form and Antibiotic Resistance

The emergence of antibiotic resistance in bacteria remains a major global health issue. Some studies have suggested a relationship between climate change and bacterial antibiotic resistance [54]. Many antibiotics currently in use, such as penicillins, cephalosporins, monobactams, vancomycin, meropenem, and piperacillin–tazobactam, target the bacterial cell wall [55–60]. Despite leading to susceptibility in in vitro susceptibility tests, these cell wall–targeting antibiotics may fail during treatment or not prevent disease recurrence [61–64]. Studies have also mentioned the occurrence of antibiotic resistance in the real world [65]. The conversion of bacteria to L-forms in adverse environments is also a potential cause of antibiotic resistance [16, 20, 27]. As indicated in Table 1, numerous experiments have demonstrated that bacteria can transition to their L-forms in the presence of antibiotics, significantly diminishing their sensitivity to these drugs [7]. In a study, ampicillin-sensitive *Sa. typhi* were exposed to ampicillin in a concentration gradient to simulate in vivo microbial exposure patterns. The results demonstrated that *Sa. typhi* exposed to gradient concentrations of the antibiotic converted to its L-form, which could cause an infection in a mouse model. The findings further suggested that the L-forms can evade immune surveillance and facilitate transmission between macrophages [13]. Aleksandra et al. reported that pathogenic *E. coli* can transform into proliferative L-forms in the presence of meropenem, and bacterial L-forms exhibit resistance to antibiotics targeting penicillin-binding proteins [7]. Georgi et al. [66] reported *Mycobacterium* L-forms obtained through low-temperature treatment are resistant to ethambutol. Furthermore, experiments by Kawai et al. demonstrated that innate immune effectors, such as lysozyme, can promote the transformation of bacteria into their L-forms [27].

**Table 1:** Experiments on drug-resistant bacterial L-forms and their results

Bacteria	Experiment	Result	Reference
*Sa. typhi*	Exposure of *Sa. typhi* to ampicillin at graded concentrations	Antibiotic gradient exposure led to can transition into L-forms, which caused infection in a mouse model	[13]
*E. coli*	Transforming *E. coli* into proliferative L-form in presence of meropenem	L-forms were resistant to antibiotics targeting penicillin-binding proteins	[7]
*Mycobacterium*	Low-temperature treatment	Ethambutol-resistant *Mycobacterium* L-forms were obtained	[6]
Gram-positive bacteria	Lysozyme treatment	L-form formation occurred	[26]
*Acinetobacter baumannii*	Treatment with a lethal concentration of meropenem	Bacteria underwent morphological changes, with numerous wall-deficient spherical cells observable under the microscope	[62]

A 2022 study investigated the persistence of *Acinetobacter baumannii* through dose-dependent antibiotic killing and found that *A. baumannii* treated with meropenem at lethal concentrations underwent morphological changes, with numerous cell wall–deficient spherical cells observed under the microscope. Subsequently, in an *A. baumannii*-infected *Galleria mellonella* model, more bacteria in the hemolymph of meropenem-treated *G. mellonella* appeared spherical compared with the untreated control group (31.2% vs. 1.4%). Thus, bacterial L-forms can form during host treatment and exhibit resistance to β-lactam antibiotics [67].

Many experiments have demonstrated that bacteria can enter the L-form state and develop resistance in the presence of antibiotics at certain concentrations in vivo. However, antibiotic resistance caused by L-forms is frequently overlooked in clinical practice [68]. This may be due to difficulties in the culture of L-forms, the longer growth and division time of L-forms compared with typical bacteria, and a lack of attention to L-forms in clinical settings [20].

To better address the challenges posed by bacterial L-forms in clinical treatment, we propose the following potential therapeutic strategies and new drug development approaches:

(1) Combination therapy using antibiotics targeting the bacterial cell membrane to either kill or inhibit bacterial L-forms. (2) Studies have shown that manidipine or flunarizine can inhibit the division of bacterial L-forms, suggesting that these drugs may be considered for treatment. (3) Development of novel, effective antibiotics targeting the bacterial cell membrane, or screening existing drugs for their ability to inhibit bacterial L-forms.

## L-form and Disease

L-forms transformation enables bacteria to evade antimicrobial agents and proliferate, and bacterial L-forms are capable of reverting to a cell-walled morphology under appropriate conditions. Thus, we propose that L-forms potentially participate in the mechanisms driving disease recurrence. Many human tissues and organs, such as the kidneys, gallbladder, and synovial fluid, meet the growth requirements for L-forms [12, 69–71].

Since the 1970s, bacterial L-forms have been isolated from patients with multiple clinical conditions, such as inflammatory bowel disease [72] and chronic gastritis [73], as well as from hemodialysis patients [74]. However, because of technological limitations, the earlier studies have lacked strong evidence. Nevertheless, as indicated in Table 2, over the past decade, some convincing studies using modern techniques have demonstrated that bacterial L-forms can be a source of recurrent infectious diseases. For instance, Mickiewicz et al. collected urine samples from 30 older patients with recurrent urinary tract infections (RUTIs) and filtered them. The filtered samples demonstrated L-form-like bacteria, and based on 16S ribosomal RNA sequencing along with 4′,6-diamidino-2-phenylindole staining, these bacterial cells were identified as *E. coli* L-forms. In a live zebrafish larvae model, the authors observed the transformation of walled cells transitioning to their L-forms under antibiotic pressure, followed by reconstitution of the cell-walled morphology after antibiotic removal [20]. Thus, bacterial L-forms may be a cause of RUTIs. Antimicrobial agents are routinely employed in the management of urinary tract infections (UTIs); Nevertheless, indiscriminate antibiotic administration can lead to elevated bacterial resistance rates, contributing to RUTIs and exacerbating patient disease burden. Therefore, identifying the causes underlying antibiotic resistance and exploring alternative therapeutic options are crucial. For instance, real-world studies have indicated that Utipro Plus, a medical device containing xyloglucan, *Sophora flavescens*, and propolis, can reduce UTI recurrence [75].

**Table 2:** Bacterial L-forms and associated diseases

Bacteria	Disease	Conclusion	Reference
*E. coli* L-form	RUTIs	L-forms may cause recurrent RUTIs	[19]
*L. monocytogenes* L-form	–	L-forms can persist in host cells, causing recurrent infections	[70]
*M. avium*	Osteomyelitis	Acid-fast spheres suggest mycobacterial L-form infection, even with a negative culture	[71]
*M. tuberculosis* L-form	Mother vaccinated with BCG	L-forms can cross the placenta	[72, 73]

RUTIs, recurrent urinary tract infections; –, none

Another study based on an in vitro cell culture model demonstrated that *L. monocytogenes* L-forms can persist within host cells as a source of recurrent infections, confirming that bacterial L-forms remain viable within host cells in vitro [76]. In 2022, a hospital in China identified the first case of osteomyelitis associated with *Mycobacterium avium* L-forms. The patient, who was HIV-positive, initially tested negative for tuberculosis according to the conventional *Mycobacterium* culture, Xpert MTB/RIF, and T-SPOT tests. However, the patient had a *Mycobacterium* infection history, and their samples demonstrated red spherical bodies after acid-fast staining; thus, a *Mycobacterium* L-form infection was suspected. Subsequent polymerase chain reaction and revertant testing confirmed that *M. avium* L-forms caused the infection [77]. A case report analysis on post-COVID syndrome also indicated that some patients developed tuberculosis symptoms after COVID-19; however, traditional bacteriological, immunohistochemical, and molecular genetic techniques failed to detect it. This was highly likely due to the bacterial cells transitioning into their L-forms [78]. In addition, *Mycobacterium tuberculosis* L-forms were detected in the lung tissue of a patient with recurrent fluoroquinolone-resistant tuberculosis. All the aforementioned cases suggested that when infection symptoms appear in combination and when conventional detection methods fail to identify *M. tuberculosis*, the bacterium should be considered to have transitioned into its L-form.

BCG-vaccinated pregnant mothers can carry *M. tuberculosis* L-forms, which can transfer to their newborns through vertical transmission; moreover, maternal BCG vaccination affects placental development [79, 80]. In a subsequent study, Markova isolated wall-deficient bacteria and fungi in blood samples obtained from autistic children and their mothers; these cells could revert to their typical morphology. In contrast, these bacteria and fungi were not found in the blood of healthy controls. Notably, wall-deficient *Aspergillus fumigatus* was detected in the peripheral blood of both autistic children and their mothers [81]. Therefore, wall-deficient bacteria or fungi may cross the placental barrier, enter the fetal environment, and establish colonization. This result provides newer insights into the causes of various congenital diseases.

In summary, numerous experimental results and case reports have indicated a strong association between L-forms and disease recurrence. Moreover, these L-forms may be linked to the etiology of several congenital disorders.

At present, numerous studies have linked bacterial L-forms with diseases, establishing a definitive association between L-forms and congenital disorders, convincing clinicians of the relationship between L-forms and disease recurrence, and identifying effective treatments for bacterial L-form infections are highly warranted.

## L-form and the Immune System

**Fig. 4:**
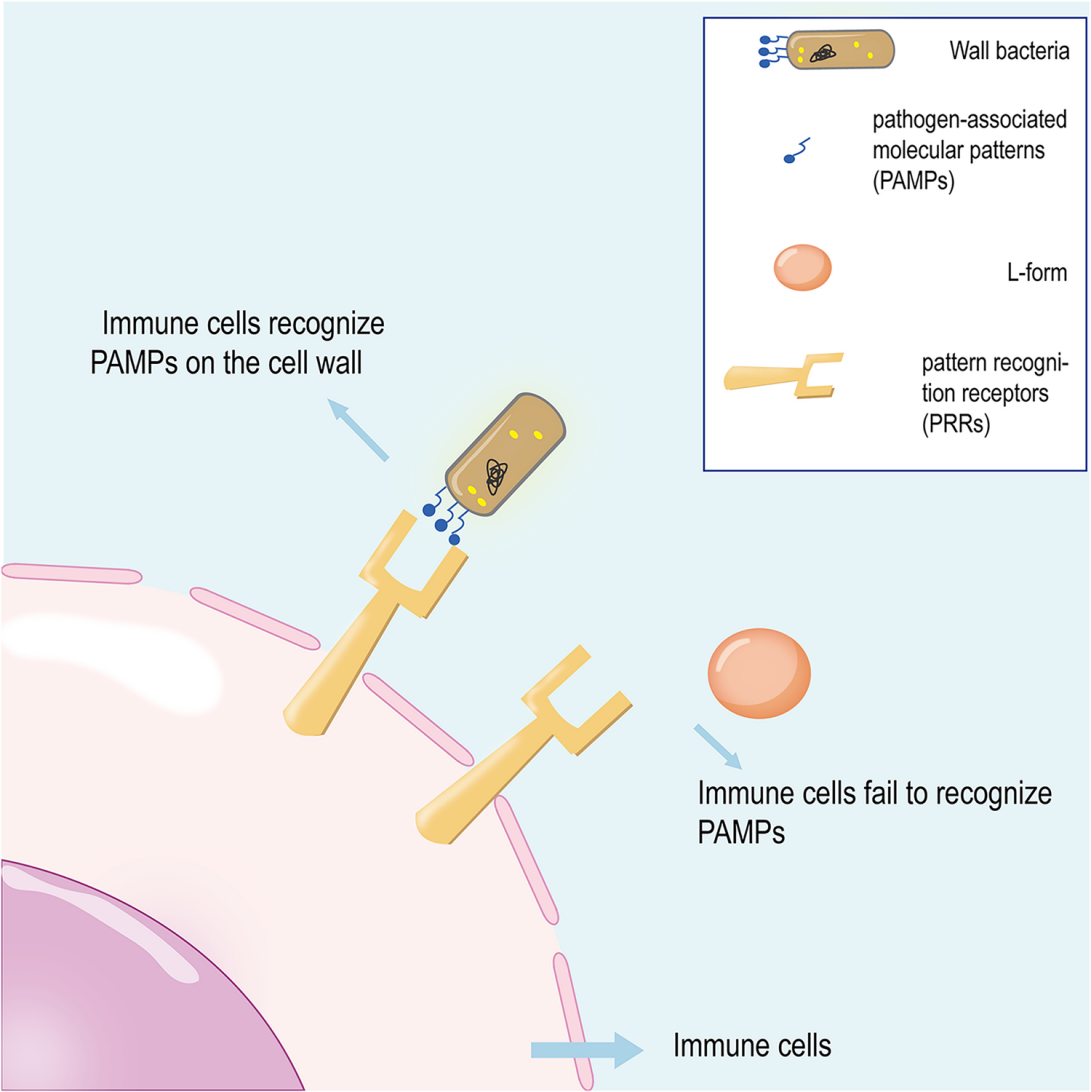
Pattern recognition receptors (PRRs) on innate immune cells specifically recognize pathogen-associated molecular patterns (PAMPs) within the cell wall components of typical bacteria, thereby initiating the immune response. However, bacterial L-forms evade innate immune recognition due to the absence of cell wall structures

There is a view that L-forms may evade recognition by the immune system. The bacterial cell wall contains various complex carbohydrates and lipids, such as lipopolysaccharides, lipoteichoic acids, and peptidoglycan. These substances serve as pathogen-associated molecular patterns (PAMPs) that can be recognized by the host’s pattern recognition receptors (PRRs). For instance, the degradation product of peptidoglycan, muramyl dipeptide (MDP), can be recognized by the NOD2 receptor. However, when bacteria transition into the L-forms, they lose their cell wall or experience impaired cell wall synthesis. Consequently, the production of PAMPs on their cell wall is significantly reduced, leading to a diminished ability of the immune system to recognize L-forms (Fig. 4).[82–85] In contrast, a more recent study suggested that the host immune system can recognize stable *L. monocytogenes* L-forms, triggering increased cytokine production. Moreover, *L. monocytogenes* L-forms may not escape phagolysosomes but may be cleared through the phagolysosomal pathway [19].

With increased frequent, improper antibiotic use, the prevalence of multidrug-resistant bacteria is increasing [86], highlighting the urgent need for new antimicrobial treatments. Bacteriophages, which can invade and lyse bacterial cells, are established as viable therapeutic agents against bacterial infections, demonstrating efficacy in numerous cases. [87–89]. However, *L. monocytogenes* can evade phage lysis by rapidly transitioning into an L-form state. This occurs because the release of lysins from bacteriophages can prompt nearby bacteria to escape. In their cell wall-deficient L-forms, these pathogens exhibit, bacteria lack the necessary ligands, such as wall teichoic acids, which phages use to recognize their hosts. Consequently, L-forms can act as a refuge, allowing bacteria to evade phage lysis. In the absence of selective pressure, however, *L. monocytogenes* L-forms revert to a walled morphology. In suitable ex vivo environments (e.g., urine), phage attacks in *Enterococcus faecium* produce outcomes similar to those observed in *L. monocytogenes* [28]. *E. coli* and *B. subtilis* can also evade phage infection by shedding their cell walls [90].

## Future Directions and Prospects for L-form Research

Although several ongoing studies and reports thus far have focused on bacterial L-forms, research interest in L-forms has waned since 2000 compared with that in the preceding period. Three main reasons, which constitute challenges demanding further investigation, underlie this decline: first, the morphological variability and difficulty in identifying bacterial L-forms; second, instability of clinically induced or antibiotic-induced L-forms, which makes subsequent research challenging; and third, a lack of awareness among clinicians that bacterial L-forms may be a reason for antibiotic resistance or treatment failure. If detectable markers related to bacterial L-forms can be identified, relevant infections could be detected easily, leading to enhanced comprehension of L-form bacteria’s relationship with antibiotic resistance, treatment failure, and false-negative culture results despite infection symptoms. Finally, bacterial L-forms may be applied in synthetic biology, including developing faster, more accurate phage therapy [91] or a novel cell fusion model [92].

## Abbreviations

UDP-GlcNAc: Uridine diphosphate *N*-acetylglucosamine
UDP-MurNAc: Uridine diphosphate-*N*-acetylmuramic acid
PG: Peptidoglycan
F-6-P: Fructose-6-phosphate
UndP: Undecaprenyl phosphate
GTase: Glycosyltransferase
Tpase: Transpeptidase
UptA: UndP transporter A
PopT: Polyprenyl-phosphate transporter
ROS: Reactive oxygen species
TCA: Tricarboxylic acid cycle
DAPI: 4′,6-diamidino-2-phenylindole
PCR: Polymerase chain reaction
RUTIs: Recurrent urinary tract infections
